# Limonene-Based Epoxy: Anhydride Thermoset Reaction Study

**DOI:** 10.3390/molecules23112739

**Published:** 2018-10-23

**Authors:** Guillaume Couture, Lérys Granado, Florent Fanget, Bernard Boutevin, Sylvain Caillol

**Affiliations:** Institut Charles Gerhardt Montpellier-UMR 5253-CNRS, Université Montpellier, ENSCM–240 Avenue Emile Jeanbrau, CEDEX 5, 34296 Montpellier, France; Guillaume.coutur@enscm.fr (G.C.); Lers.granado@enscm.fr (L.G.); flrorian.fanget@enscm.fr (F.F.); Bernard.boutevin@enscm.fr (B.B.)

**Keywords:** limonene, biobased polymers, anhydride, epoxy, DSC

## Abstract

The development of epoxy thermosets from renewable resources is of paramount importance in a sustainable development context. In this paper, a novel bio-based epoxy monomer derived from limonene was synthesized without epichlorohydrine and characterized. In fact, this paper depicts the synthesis of bis-limonene oxide (bis-LO). However, intern epoxy rings generally exhibit a poor reactivity and allow reaction with anhydride. Therefore, we used a reaction model with hexahydro-4-methylphthalic anhydride to compare reactivity of terminal and interepoxy functions. We also studied the influence of methyl group on intern epoxy functions. Furthermore, the influence of epoxy:anhydride stoichiometry and initiator amount was studied. These studies allow to propose an optimized formulation of bis-LO. Finally, a bis-LO-based thermoset was obtained and characterized.

## 1. Introduction

With the decrease of easily available fossil resources, ensuing price volatility, and the increasing awareness regarding environmental and human health protection, a booming amount of research has been carried out to find alternative to fossil-based chemicals. This statement is especially true for thermosetting polymers (or thermosets), which represent about 20% of the global plastic production with a large number of applications. In fact, thermosets, as crosslinked nonfusible three-dimensional networks, cannot be recycled like most of thermoplastic materials [1]. Thus, the synthesis of thermosets from bio-based resources appears to be of upmost importance. 

**Scheme 1:**
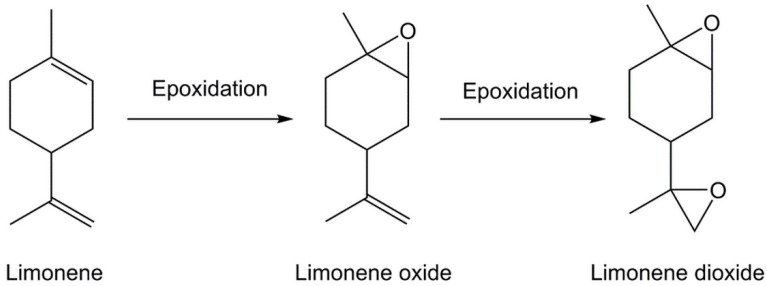
Synthesis of limonene oxide and limonene dioxide from limonene.

Among the various thermosets designed at an industrial scale, polyepoxides account for 70% of the market thanks to their high mechanical properties, chemical and moisture resistance or easy processability [2]. Unfortunately, around 90% of them are based on diglycidylether of bisphenol A (DGEBA), an oil-based molecule obtained from bisphenol A (BPA). BPA has been classified as carcinogen mutagen and reprotoxic (CMR). Moreover this is an endocrine disruptor which is submitted to a more and more restrictive regulation in numerous countries [3]. An increasing number of academic and industrial researches have therefore been dedicated to find bio-based, nontoxic alternatives to BPA for the synthesis of original epoxy networks [1,3,4] such as: vanillin and its derivatives [5,6,7,8], eugenol [9], ferulic acid [10], isosorbide [11,12], catechin [13], cardanol [14,15], and vegetable oils [16,17]. Similarly, most epoxy networks are synthesized from epichlorohydrin, which is a CMR and a highly toxic molecule. Alternatives have thus been considered, especially via the epoxidation of preexisting carbon–carbon double-bonds. Among potential bio-based molecules, limonene (Scheme 1) appears as an interesting candidate. It is a cyclic monoterpene derivative found in many citrus fruits [18]. The latter are the most abundant tree crops with a global production of 88 million tons per year and include oranges and lemons among others. Around 50% of theses fruits are processed into juice or marmalade, yielding an estimated 40 million tons of citrus waste world-wide [19]. Depending on the fruit variety, season and geographic origin, a variable amount of limonene can be extracted, generally accounting for 4% *w*/*w* of the citrus waste, for an estimated production of 77 thousand of tons per year [20].

The carbon–carbon double bond located on the aliphatic ring of limonene can be epoxidized to yield limonene oxide (Scheme 1) by various methods, generally involving organic peroxides, peroxy acids or recently hydrogen peroxide in the presence of enzymes [21]. The main advantage of this route lies in the absence of epichlorohydrin. Further epoxidation can also be carried out to obtain limonene dioxide. However, the difference in reactivity of its two epoxy groups and the limited tunability of limonene dioxide-based networks decrease its suitability for thermosets synthesis. More surprisingly, to the best of our knowledge, only one recent paper described the use of limonene oxide for this application. In fact, Morinaga and Sakamoto [22] synthesized multifunctional limonene-based epoxides using di-, tri- or tetra-thiols and crosslinked them with branched polyethyleneimine. They observed that a large excess of amine groups was required to obtain satisfactory crosslinking at 100 °C. 

Very few other epoxy networks were synthesized with cycloaliphatic epoxides [23,24,25]. Recently, Wang and Schuman [25] crosslinked blends of DGEBA and vegetable-oil-derived epoxy monomers with hexahydro-4-methylphthalic anhydride (HMPA), creating the first partially bio-based epoxy thermoset obtained using cyclic anhydrides. Usually, epoxy monomers are crosslinked by toxic, oil-based polyamines, but according to Yang et al. [26], anhydrides provide epoxy thermosets with lower toxicity and higher glass transition temperature. Furthermore, Buchmeiser et al. proposed innovative approach by using protected *N*-heterocyclic carbenes as latent organocatalysts for the low-temperature curing of epoxy:anhydride thermosets [27,28]. Moreover, it is possible to obtain a one-component mixture with an increased pot-life with epoxy/anhydride systems [29] Anhydrides are potentially bio-based [30,31] and can provide the thermosets with tunable properties.

**Scheme 2:**
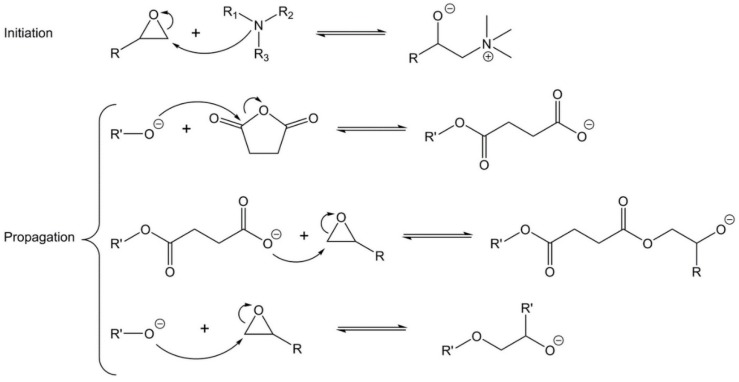
Curing mechanism of succinic anhydride and epoxide initiated by a tertiary amine.

**Scheme 3:**
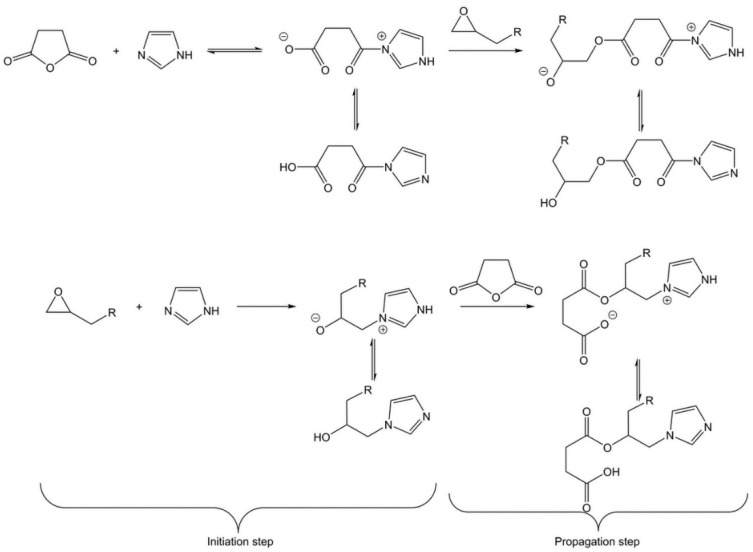
Curing mechanism of succinic anhydride and epoxides initiated by imidazole.

The mechanism of curing of epoxy with anhydrides differs from amines since it exhibits initiation, propagation and termination steps similar to a ring-opening polymerization. Conflicting initiation mechanisms have been reported, but most of them agree on the formation of a zwitterion [29,32,33,34]. For Park et al. [32], it occurs via the ring-opening of the epoxide by the catalyst (Scheme 2), while the nucleophilic attack can similarly occur on the anhydride according to Amirova et al. [29] (Scheme 3). Antoon and Koenig [35] describe an amine-catalyzed isomerization of the epoxy groups into unsaturated alcohols followed by the formation of a ternary complex reacting on another epoxy (Scheme S1 in Supporting Information). The formed anionic active centers then go through the propagation steps: alkoxide anions react with anhydrides to yield a mono-ester carboxylate that reacts with another epoxy ring. However, Park et al. mention that alkoxide anions can simultaneously react with other epoxides to yield ether instead of ester bonds (Scheme 2). Finally, termination step occurs by deactivation of the active center.

Despite these complex and interesting mechanical studies, few information was given regarding the influence of stoichiometry and catalyst amount on thermosets, a trend also observed when DGEBA was cured with anhydrides [27,30]. Park et al. [30] mentioned a 1:1 epoxy:anhydride formulation ratio with epoxy in excess and observed slightly higher activation energy for the epoxy-rich formulations as well as a visible etherification peak on DSC curves. Matejka et al. [36] and Steinmann [37] studied the influence of various experimental parameters on epoxy-anhydride linear polymers. Steinmann [38] carried out the curing of DGEBA with phthalic anhydride and studied the influence of catalyst concentration on the *T_g_* of the thermosets. She stated that the highest glass transition temperatures were obtained at different initiator concentrations depending on its nature, ranging between 0.05 and 0.1 mol%. Recently, Paramarta and Webster [39] studied the influence of stoichiometry and catalyst amount on the kinetics parameters of the curing reaction but did not provide thermal or mechanical properties. Finally, Kuncho et al. [40] noticed a maximum hardness for epoxidized linseed oil/HMPA/1,8-diazabicyclo[5.4.0]-undec-7-ene materials with 1:1 epoxy:anhydride ratio. They also observed an initial increase in hardness followed by a plateau when increasing the initiator amount. Additionally, Schultz et al. demonstrated by Matrix Assisted Laser Desorption Ionisation-Time of Flight (MALDI TOF) the perfect alternating structure of epoxy:anhydride thermosets [41].

**Figure 1:**
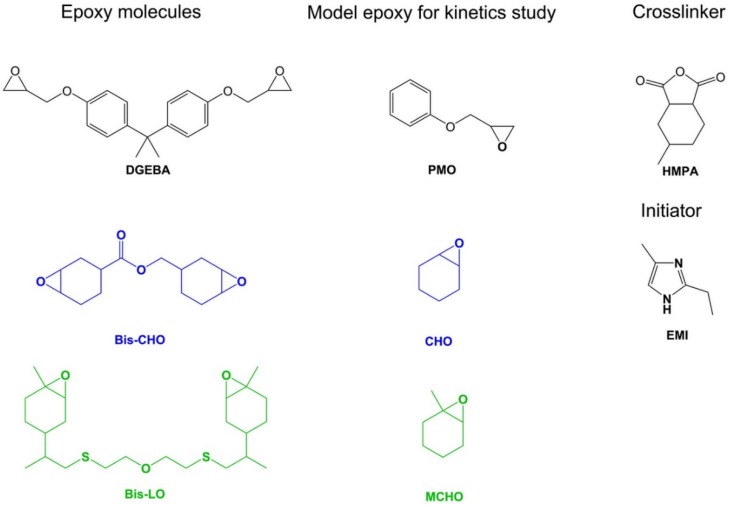
Chemical structure and abbreviations of the model molecules, diepoxides, curing agent and initiator catalyst.

Thus, the first aim of this paper is to further enhance the knowledge on cycloaliphatic epoxy/anhydride curing systems and the possibility to use bio-based cyclo-aliphatic epoxy monomers as precursors for thermosets materials. Indeed, cycloaliphatic epoxides could be directly obtained—without epichlorohydrin—from some terpenes, an important class of renewable resources. Hence, in a first part, the synthesis of bis-limonene oxide (bis-LO) is presented. Then, we compare the reactivity of epoxies DGEBA, bis-cyclohexene oxide (bis-CHO) and bis-LO with the help of model epoxies molecule phenyl glycidyl ether (PMO), cyclohexene oxide (CHO) and 3,4-epoxycyclohexylmethyl (MCHO), respectively (Figure 1). Noncrosslinking model molecules are chosen rather than thermosets to get free from the effect of formulations and to avoid unwanted variation of the kinetics due to the gelation and vitrification (occurring during crosslinked network formation). Hence, the influence of the various functions close to the epoxy moiety during the curing behavior can be clarified. The glycidyl epoxy position is compared to cycloaliphatic epoxy (PMO and CHO), the influence of methyl group on cycloaliphatic epoxy is evaluated (CHO and MCHO). The second part focuses on the influence of stoichiometry and initiator amount of thermo-mechanical behavior of model thermosets based on DGEBA and bis-CHO. This analysis is used to propose an optimized formulation of bis-LO with HMPA. Finally, the thermal resistance of the bis-LO is compared to model formulations by TGA. 

## 2. Experimental Part

### 2.1. Materials

Cyclohexene oxide (98%), (+)-Limonene oxide, mixture of *cis* and *trans* (97%), 3,4-epoxycyclohexylmethyl 3,4-epoxycyclohexanecarboxylate, 2-ethyl-4-methylimidazole (95%), hexahydro-4-methylphthalic anhydride, mixture of *cis* and *trans* (96%), phenyl glycidyl ether (96%), diglycidyl ether of bisphenol A (DGEBA), and ethyl acetate were supplied by Sigma-Aldrich (Saint-Louis, MO, USA). Bis(2-mercaptoethyl)ether (95%) and 2,4,6-trimethylphenol (98%) were supplied by ABCR (Karlsruhe, Germany). Deuterated DMSO (DMSO-d6) (99.8%D) was supplied by Euriso-Top (Saint-Aubin, France). Azobisisobutyronitrile was supplied by Fluka and purified by recrystallization in methanol. All other reagents were used as received.

### 2.2. Synthesis of Bis-LO

In a 250 mL round-bottom flask with a magnetic stirrer, limonene oxide (20.00 g, 0.131 mol), azobisisobutyronitrile (2.00 g, 1.22 × 10^−2^ mol i.e., 10 wt.% compared to limonene), 40 mL of ethyl acetate and bis(2-mercaptoethyl) ether (8.90 g, 6.44 × 10^−2^ mol i.e., 0.49 molar equivalents of limonene) are inserted and heated at 60 °C for 16 hours. After cooling at room temperature, the crude product is poured into a separating funnel and 40 mL of a 2 M NaOH aqueous solution are added. The aqueous phase is washed with 40 mL of ethyl acetate and the total organic phase is washed with 40 mL of deionized water and 40 mL of brine. After drying over MgSO_4_, the solvent was removed in a rotary evaporator and the product was dried overnight, in an oven at 40 °C under reduced pressure (1 × 10^−1^ mbar). Product appears as an uncolored, transparent, slightly viscous liquid.

### 2.3. Thermoset Curing

The epoxy monomer, HMPA and 2-ethyl-4-methylimidazole (EMI) were weighed, poured into an aluminum pan and stirred manually for 5 min prior to heating in an oven at the desired temperature. Bis-CHO-based thermosets were obtained after 4 h at 180 °C followed by 1 h at 190 °C, while their DGEBA-based counterparts were crosslinked at 120 °C for 4 h and 1 h at 140 °C.

### 2.4. Characterization Techniques

The Nuclear Magnetic Resonance (NMR) spectra were recorded on a Bruker AC 400 instrument (Billerica, MA, USA), using deuterated chloroform or dimethylsulfoxide-*d*_6_ as solvents. 

Attenuated total reflectance Fourier transform infrared absorption spectroscopy (ATR-FTIR) experiments were carried out on a NICOLET 6700 spectrometer from Thermo-Scientific (Waltham, MA, USA), equipped with a mercury-cadmium-tellurium (MCT) detector, in the middle infrared range with a resolution of 4 cm^−1^ and 32 scans were added to each spectrum.

Differential scanning calorimetry (DSC) experiments were carried out on a DSC200 F3 Maia from Netzsch (Selb, Germany), equipped with an intra-cooler system and under nitrogen environment. The DSC was calibrated with biphenyl, indium, bismuth and CsCl highly pure standards, at 10 °C/min. Samples were weighed into stainless-steel crucibles sealed with a gold seal for kinetics studies. DSC was also used to determine the glass transition temperatures of the cured thermosets (Al pan and pierced lids).

Thermo-gravimetric analysis (TGA) experiments were recorded on a TGA Q50 from TA instruments using a platinum crucible. The samples were heated up from 20 to 800 °C at 20 °C/min, under nitrogen atmosphere.

## 3. Results and Discussion

### 3.1. Synthesis of Bis-Limonene Oxide (Bis-LO)

**Scheme 4:**
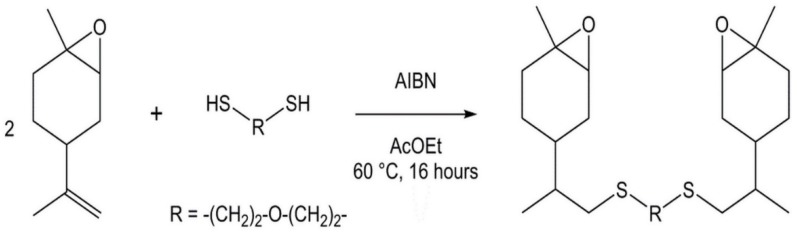
Synthesis of bis-limonene oxide using thiol-ene coupling.

Eugenol is a bio-based phenolic derivative exhibiting a carbon–carbon double-bond. Through functionalization with epichlorohydrin, it yields glycidylated eugenol that has been reacted by Zou et al. [42] with various di-thiols via a thiol-ene coupling to synthesize shape memory epoxy networks. Considering the similarities between glycidylated eugenol and limonene oxide, bis-limonene oxide derivatives have been synthesized following a similar pathway using azobisisobutyronitrile (AIBN) as a source of radicals (Scheme 4). Limonene oxide was used in slight excess to ensure a maximum yield of bis-epoxides. After purification steps, the obtained products were characterized by FTIR and NMR spectroscopies.

**Figure 2:**
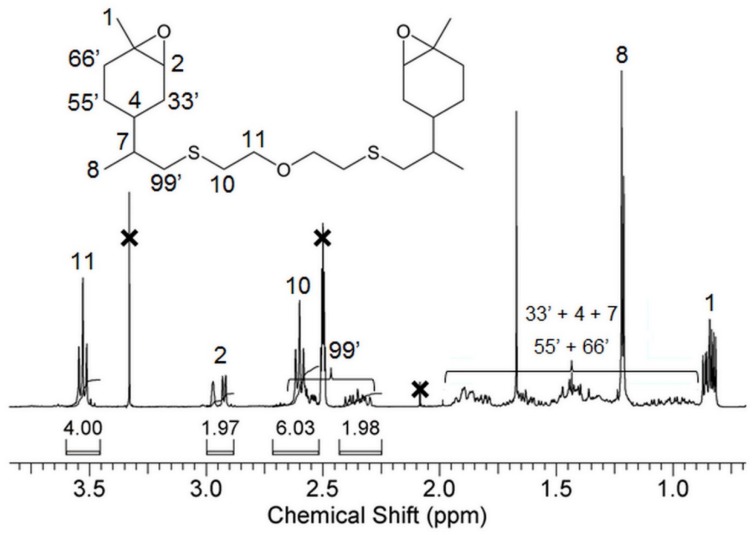
^1^H-NMR spectrum of bis-LO recorded in deuterated DMSO.

The analysis of the bis-LO by FTIR spectrum shows the disappearance of the C=C double bond signals at 884 cm^−1^ (C=C out-of-plane bending), 1645 cm^−1^ (C=C stretching) and 3072 cm^−1^ (=C-H stretching) when compared to limonene oxide (see Figure S1 in Supporting Information). All other signals of bis-LO correspond to a combination of the characteristic signals of both limonene oxide and bis(2-mercaptoethyl) ether such as C-H bonds stretching between 2780 and 3010 cm^−1^ and the C-O bond stretching of the ether groups at 1100 cm^−1^. These encouraging results were confirmed by ^1^H-NMR spectroscopy (Figure 2). Similarly to FTIR spectroscopic data, the signals of the limonene oxide C=C double bond protons at 4.6–4.7 ppm disappeared after the reaction, indicating a high conversion of limonene oxide towards the thiol-ene coupling. The signals shifted at 2.3–2.4 ppm and 2.5–2.6 ppm (**9** and **9′** on Figure 2) in the form of multiplets because of the asymmetric carbons C_E_ and C_H_ (see Figure S2 in Supporting Information). The latter also overlaps with the triplet of the -CH_2_-S protons (**10**) from grafted bis(2-mercaptoethyl) ether at 2.6 ppm. A similar triplet can be observed at 3.5 ppm that corresponds to the -CH_2_-O protons (**11**). The methanetriyl proton (**7**) that could also confirm the grafting unfortunately overlaps with all the other aliphatic signals between 0.8 and 2.0 ppm. Integrations highlight the efficiency of the synthesis and the absence of possible side-reactions such as epoxy ring opening from thiols. All these signal assignments were confirmed by ^13^C, HSQC ^1^H-^13^C- and HMBC ^1^H-^13^C-NMR spectroscopies (Figures S2–S4, respectively).

For the purpose of limiting the number of costly purification steps regarding the potential applications of bis-limonene oxide derivatives, no chromatographic separation was carried out on bis-LO. Thus, the mixture might contain some traces of unreacted limonene oxide (although not observed by NMR spectroscopy) and isobutyronitrile from AIBN recombination. The epoxy content of bis-LO was therefore determined by NMR dosing using 2,4,6-trimethylphenol as an external standard. The signal of the methanetriyl proton (**2**) at 2.8–3.0 ppm was used as the reference in Equation (1).

(1) $$Epoxy\;Equivalent\;\left(mol/g\right)=\frac{m}{\frac{m_{S}}{M_{S}}*\frac{2*\int_{2.8}^{3.0}CH_{epoxy}}{\int_{6.7}^{6.9}CH_{S}}}$$

With *m* and *m_S_* the mass of the resin and the standard (in g), respectively, *M_S_* the molecular weight of the standard (in g mol^−1^). A value of 4.40 ± 0.08 mmol⋅g^−1^ (equivalent weight of epoxy mixture of EEW = 227 g/eq) were obtained for bis-LO and used for further materials formulations.

### 3.2. Kinetics Study on Model Molecules 

**Figure 3:**
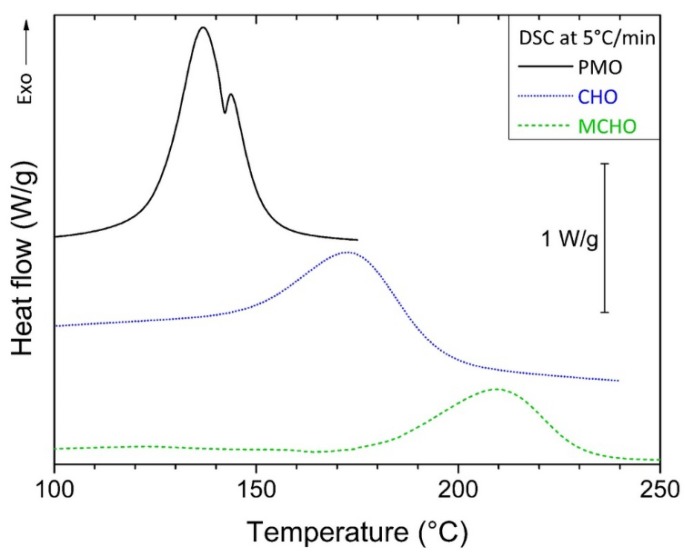
DSC thermograms of PMO, CHO and MCHO at 5 °C/min.

The reaction between model epoxies (PMO, CHO and MCHO) and anhydride (2/1 *n*/*n*) in presence of initiator (1 wt%) is monitored by DSC, with nonisothermal programs, at constant heating rates (*β* = 3, 5, 8, 10 °C/min). Typical thermograms at 5 °C/min are presented in Figure 3, for the three studied systems. In all cases, exothermic signals are recorded, as expected for epoxy ring opening reactions. For PMO two peaks are observed (near 135 and 145 °C), whereas one single peak is present for CHO and MCHO. The first PMO peak is assigned to the reaction between epoxy and anhydride. The second peak corresponds to the reaction between two epoxy rings (i.e., homopolymerization). Notably, the reaction between HMPA and PMO lead to a fine and intense exothermic peak, whereas with CHO and MCHO the peak is much broader and less intense.

The reactivity of these model molecules can be readily appreciated by monitoring the onset of the exothermic peak (Table 1). For example, at 5 °C/min (Figure 3), the peak onsets are 126, 149 and 174 °C for PMO, CHO and MCHO, respectively. These significant differences lead to conclude in a trend of reactivity of the studied epoxy models with HMPA. Hence, glycidyl epoxy (PMO) is more reactive than cycloaliphatic epoxy (CHO) and even more thermal energy is required to enable the reaction ring opening of cycloaliphatic methyl-epoxy (MCHO).

In addition, it is interesting to compare the reaction enthalpy values (Table 1). CHO presents higher enthalpy per mass unit values (318.5 ± 6.8 J/g) compared to PMO (278.6 ± 12.9 J/g) and MCHO (182.0 ± 24.3 J/g). However, when the reaction enthalpy is calculated regarding the amount of epoxy, a different trend is observed. CHO and PMO present sensibly similar values (58 and 62 kJ/mol, respectively). These values are in accordance with literature data [43]. In contrast, the MCHO value of 36 kJ/mol is two times lower, suggesting again a lower reactivity of the methylated-epoxy groups.

The conversion of the reaction (*x*) between model epoxies and anhydride is calculated by integrating DSC thermograms (with linear baseline approximation), with the following equation:

(2) $$x\left(t,T\right)=\;\frac{\Delta H_{t,T}}{\Delta H_{TOTAL}}$$

where $\Delta H_{t,T}=\;\int_{0}^{t}\dot{q}\left(t,T\right)dt$ is the cumulative released heat of reaction at the time *t* and curing temperature *T*, as time integral of the instantaneous heat flow ($\dot{q}\left(t,T\right)$), and $\Delta H_{TOTAL}$ is the total enthalpy of the reaction (total area). 

**Figure 4:**
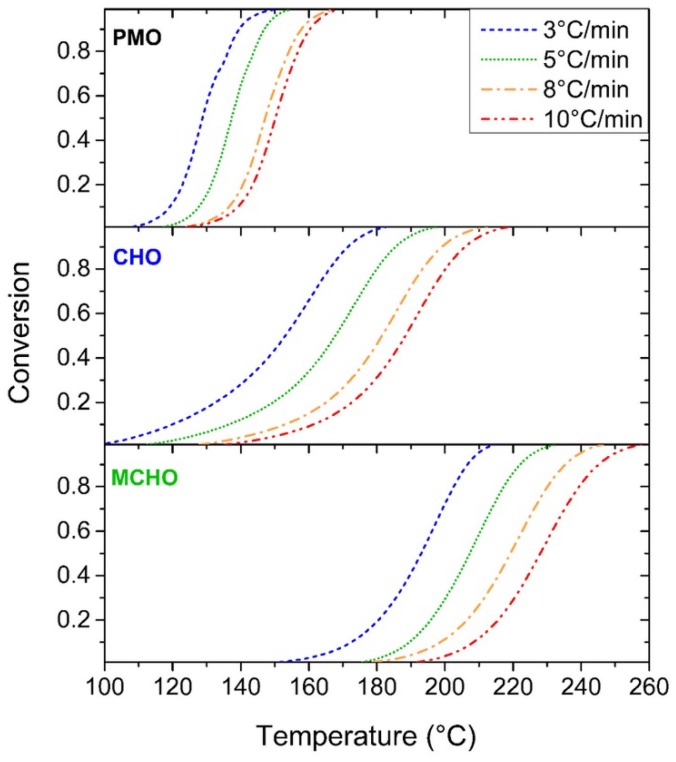
Nonisothermal kinetic profiles of PMO, CHO, and MCHO at 3, 5, 8 and 10 °C/min.

The nonisothermal kinetic profiles of PMO, CHO and MCHO are presented in Figure 4. The conversions (Equation (2)) are plotted against the temperatures, for different linear heating rates. The curves exhibit an expected sigmoidal shape. Notably, CHO and MCHO profiles are spread on a wide range of temperature (about 100–210 °C and 150–260 °C, respectively), whereas PMO profiles are restrained at lower temperature between 110 and 170 °C. The kinetic profiles follow the previous observation, PMO reaction occurs at lower temperature than CHO, and MCHO reaction proceeds at the highest temperature range. 

In order to evaluate the activation energy of the reaction between HMPA and PMO, CHO and MCHO, isoconversional analysis is performed on nonisothermal kinetic profile datasets (integral method). Isoconversional analysis is a model-free kinetics approach, which relies on the variable separation between conversion, *x*, and temperature, *T*, leading to the equation [44]:

(3) $$\frac{dx}{dt}=k\left(T\right)\cdot f\left(x\right)$$

where *dx/dt* is the reaction rate, *k* is the rate constant and *f* is a function of the conversion (representing the reaction mechanisms). The rate constant is assumed to be described by the Arrhenius equation:

(4) $$k\left(T\right)=A\cdot e^{-\frac{E}{RT}}$$

where *A* is the pre-exponential factor, *E* the activation energy, *R* the gas constant. Because *x* and *T* are considered independent variables (i.e., the temperature schedule does not influence the reaction mechanisms), the activation energy can be determined as follows:

(5) $${\left[\frac{\partial \ln\left(dx/dt\right)}{\partial T^{-1}}\right]}_{x}=\;{\left[\frac{\partial \ln\left(k\left(T\right)\right)}{\partial T^{-1}}\right]}_{x}+{\left[\frac{\partial \ln\left(f\left(x\right)\right)}{\partial T^{-1}}\right]}_{x}=\;-\;\frac{E_{x}}{R}$$

It is to be noted that one activation energy, *E_x_*, is associated to one conversion, *x*. Hence, it is common to find variable activation energy with *x*, in isoconversional analysis.

In this study, isoconversional analysis is implemented using Vyazovkin integral method (VA). The detail of VA is very well described elsewhere [45]. In summary, VA relies on the following function minimization:

(6) $$\min\Phi \left(E_{x}\right)=\;\overset{n}{\underset{i=1}{\sum }}\underset{j\neq i}{\sum }\frac{\;I_{i}\left(E_{x},\;T_{x,i}\right)}{\;I_{j}\left(E_{x},\;T_{x,j}\right)}$$

where indexes *i* and *j* represents two heating rates curves (Figure 3) and *I* are the temperature integrals:

(7) $$I_{i}\left(E_{x},\;T_{x,i}\right)=\;\beta_{i}^{-1}\int_{T_{x-\Delta x}}^{T_{x}}e^{-\;\frac{E_{x}}{RT_{x,i}}}\cdot dT$$

where *β_i_* is the *i*th heating rate. In fine, *E_x_*is the result of the function minimization associated to one conversion, *x.* The conversions are considered from *x* = 0.01 to *x* = 0.99, with Δ*x* = 0.01, for the present implementation. It is important to stress that the activation energy values found with isoconversional analysis are apparent values. They may therefore arise from different contributing phenomena (e.g., competing reactions, diffusional restriction barriers).

**Figure 5:**
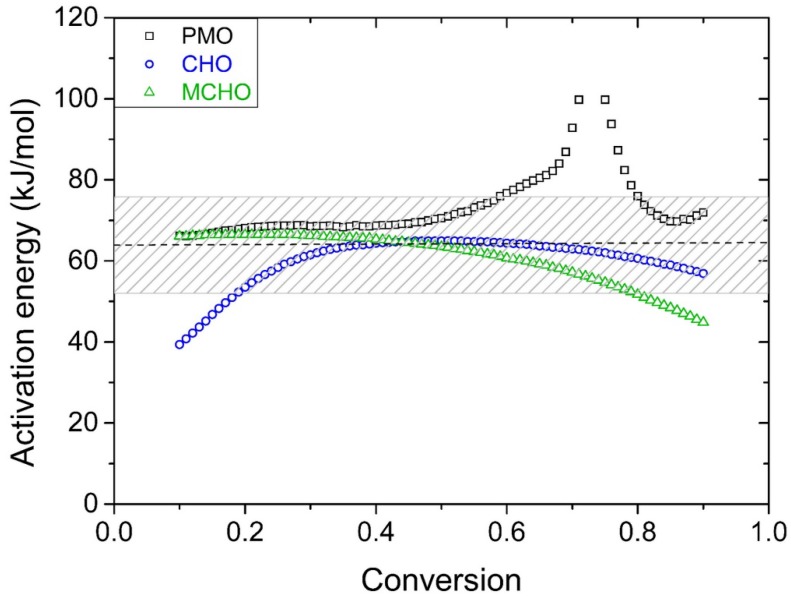
Activation energy as a function of the conversion between HMPA and PMO, CHO and MCHO, as calculated with VA method.

The results of the VA method are presented in Figure 5. The activation energy is considered as a function of conversion. For the cases of PMO and MCHO reactions, the activation energy is constant for *x* < 0.4 (*E* = 68 kJ/mol). On the other hand, the activation energy increases slightly in a first regime for CHO. This increase is assigned to uncertainties linked to integration in early conversion data points. Then the trend of PMO curve differs from CHO and MCHO. CHO and MCHO curves exhibit a decreasing trend for *x* > 0.4. In contrast, the sharp increase of PMO curve is assigned to inaccuracies of calculations. Isoconversional analysis is unable to discriminate the reaction between HMPA and PMO, and PMO on itself, leading to an over-evaluation of the values of activation energy. Nonetheless, sufficiently far from *x* ∼ 0.7 (conversion at which PMO starts to react on itself), the activation energies of HMPA/PMO (for *x* < 0.4) and homopolymerization PMO/PMO (*x* > 0.8) reactions can be respectively evaluated. 

In overall, all curves are interestingly similar. These results allow concluding that the activation energies of glycidyl, methylated- and neat-cycloaliphatic epoxies are sensibly similar, near 64 ± 12 kJ/mol. This value is slightly above previous literature data [46,47]. Furthermore, the trends of CHO and MCHO curves offers interesting insights for the reaction between these cycloaliphatic epoxies and anhydride. The decreasing trend of activation energy suggests that the reaction is autocatalytic. The reaction products may self-catalyze the reaction, such as OH moieties as products of anhydride opening (as similarly described for epoxy-amine systems [48]). 

### 3.3. Influence of Stoichiometry and Initiator Amount on T_g_

For epoxy/amine systems, the stoichiometry is known to display a crucial contribution in the three-dimensional network building and thus on the final glass transition temperature (*T_g_*) of thermosets. Therefore the stoichiometry needs to be optimized to obtain a chemically and mechanically resistant material, with the highest glass transition temperature [5]. Nevertheless, to the best of our knowledge, no such rule has been established for epoxy/anhydride reaction. As follows, we propose to clarify the impact of the epoxy/anhydride ratio and the amount of the catalyst on the glass transition temperature, for two thermoset models bis-CHO/HMPA and DGEBA/HMPA, in presence of EMI as catalyst. All experimental data are gathered in Tables S1 and S2, and DSC thermograms are displayed on Figures S5 and S6 in Supporting Information.

**Figure 6:**
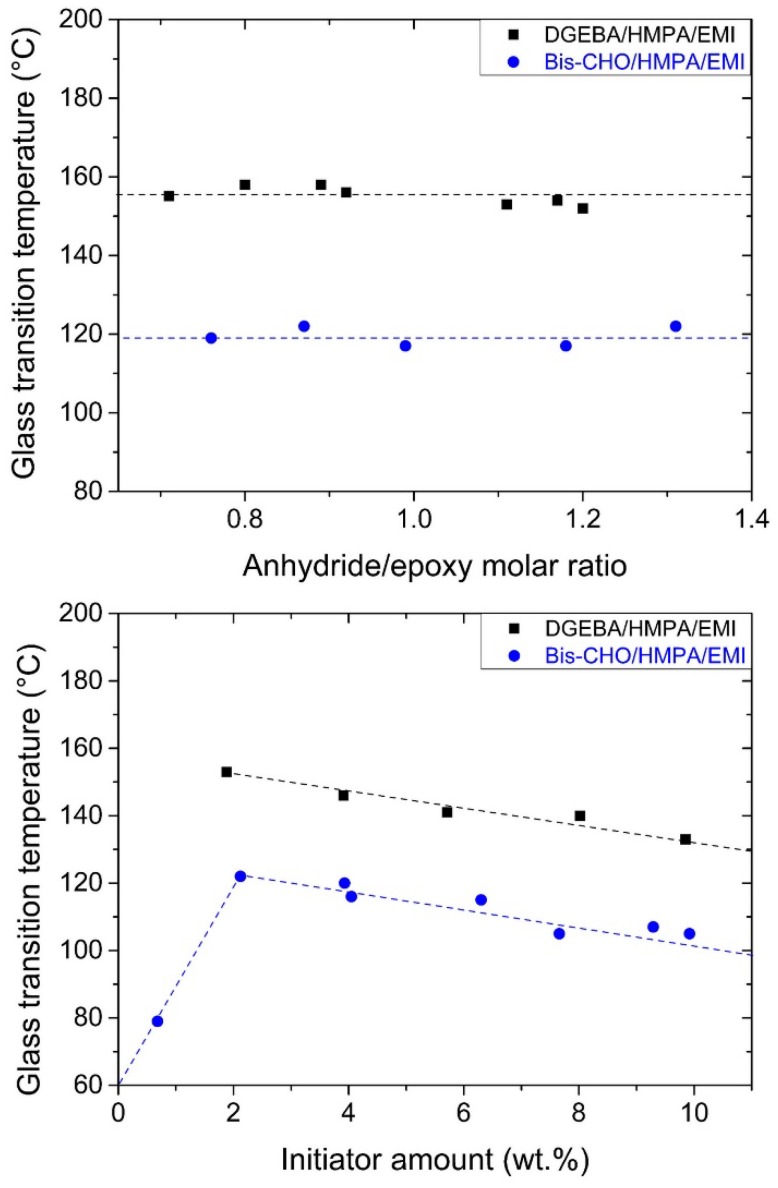
Influence of (**top**) thermoset stoichiometry and (**bottom**) initiator amount on glass transition temperature, for Bis-CHO/HMPA/EMI and DGEBA/HMPA/EMI thermosets.

The influence of the stoichiometry on *T_g_* is reported in Figure 6 (top). The glass transition temperature of the materials was determined by DSC on cured formulations (Figure S5). *T_g_* of DGEBA formulations are found near 158 °C, whereas glass transition temperatures are lower for bis-CHO formulations (*T_g_* ∼ 120 °C). The higher *T_g_* of DGEBA formulations is explained by the presence of aromatic rings that confer higher rigidity to the three-dimensional polymer network and limit mobility of network. Additionally, the sulfur hinges confer added mobility to aliphatic chains, which contributes also to lower *T_g_* value. Hence, the network needs less thermal energy to relax with cycloaliphatic epoxy, compare to aromatic-based ones. Interestingly, the glass transition temperatures are found to be constant over a rather wide range of anhydride/epoxy ratios, from 0.7 to 1.4, for both aromatic (DGEBA) and cycloaliphatic (bis-CHO) epoxies. Therefore, these results suggest that the stoichiometry does not strongly impact the glass transition temperature of final materials in epoxy/anhydride systems (unlike epoxy/amine). 

In addition, the influence on the initiator amount is shown in Figure 6 (bottom). As previously described, the *T_g_* of DGEBA formulations are globally higher than bis-CHO ones (ca. + 35 °C). For initiator amounts higher than 2 wt.%, the *T_g_* of both formulations linearly decreases from 158 to 142 °C for DGEBA and from 122 to 105 °C for bis-CHO. Interestingly, the slopes of the curves are rather similar. These decreases in glass transition is assigned to plasticizing effect of the EMI into the polymer network. Furthermore, the *T_g_* of bis-CHO formulation decreases, for [EMI] < 2 wt.%. Hence, all reactive moieties are not activated at low amount of initiator, leading to incomplete crosslinking. This cannot be studied on DGEBA. The dangling chains, containing the unreacted epoxies and anhydride groups, act as plasticizers and contribute to lower the glass transition temperature. 

In overall, it has been shown that stoichiometry has not a strong effect on the glass transition temperature of the final crosslinked materials. In opposition, the amount of initiator displays a greater role in the thermo-mechanical behavior. An optimum amount of EMI as initiator is determined from Figure 6, at ca. 2–2.5 wt.%. These findings would be of a precious help for further development of other epoxy/HMPA systems. 

Consequently, a 1:1 ratio for epoxy:anhydride and an initiator amount of 2.5 wt.% were chosen for the formulation of bis-limonene oxide networks with HMPA (bis-LO). After curing (4 h at 180 °C) and post-curing (1 h at 190 °C), the material was solid and presented a moderate glass transition temperature (*T_g_* = 75 °C).

### 3.4. Thermal Degradation Behavior

**Figure 7:**
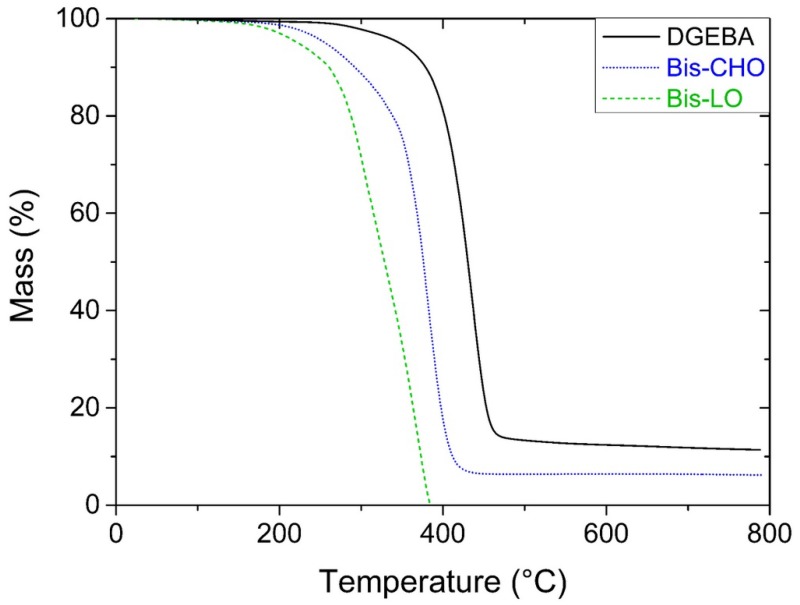
TGA thermograms of DGEBA, bis-CHO and bis-LO based networks with HMPA.

Thermal resistance of the bis-LO formulation was evaluated by TGA, in comparison to DGEBA and bis-CHO model formulations (Figure 7). As expected, DGEBA formulation exhibits higher thermal resistance than bis-CHO and bis-LO (temperature at 10% of degradation (*T_d10%_*) of DGEBA is 379 °C against 292 and 261 for bis-CHO and bis-LO, respectively). This thermal behavior of DGEBA is mainly due to the higher aromatic density. Bis-CHO exhibits rather better performances than bis-LO, probably because of the thioether-ether aliphatic chains between the epoxies in bis-LO. In fact, the relative small units in bis-CHO may results in denser crosslinked network, and thus enhancing the thermal resistance. In overall, the thermal behavior of bis-LO is relatively satisfying, as a green alternative of petroleum-based epoxy resins.

## 4. Conclusions

In this paper, we studied parameters that could influence reaction between anhydride and cycloaliphatic epoxide monomers. This reaction was much debated in literature and we showed the influence of reactants stoichiometry and initiator quantity on *T_g_* value of network, which is an important parameter. Cycloaliphatic epoxides could be very interesting monomers directly obtained—without epichlorohydrin—from some terpenes, an important class of renewable resources. Hence, we extended our study to a novel approach to synthesize a limonene-based epoxy monomer, without the use of epichlorohydrin. We performed synthesis at the lab-scale with 20 g of limonene, but the epoxidation route could easily be upscaled, since this is the route which is currently performed at Industrial scale for the epoxidation of plant-oils. Formulation was performed with hexahydro-4-methylphthalic anhydride to yield thermosets with high glass transition temperatures. This study allowed to compare reactivity of terminal and intern epoxy rings, as well as influence of methyl group on intern epoxy ring. This paper reports the influence of epoxy:anhydride stoichiometry and initiator amount that were previously much debated in literature. If the epoxy:anhydride stoichiometry has a low influence on a large range of ratios, the initiator amount has an impact on thermomechanical properties. The properties of the obtained materials were compared to the petroleum-based epoxy (DGEBA) cured with the same anhydride. This new bio-based thermoset exhibits good thermomechanical properties which allow to present limonene as a promising molecule for further studies for BPA replacement in some applications. 

## Figures and Tables

**Table 1:** Enthalpies of reaction and exothermic peak onsets as a function of the heating rates, for PMO, CHO and MCHO.

*β* (°C/min)	Δ*H_TOTAL_* (J/g)			Peak Onset (°C)		
	PMO	CHO	MCHO	PMO	CHO	MCHO
3	287.9	312.8	214.4	118	137	170
5	284.9	320.8	170.0	126	149	174
8	281.9	327.1	185.3	136	160	193
10	259.5	313.1	158.1	137	166	201
Averages	278.6	318.5	182.0			
Deviations	12.9	6.8	24.3			

## Supplementary Materials

The following are available online. Scheme S1: Curing mechanism of succinic anhydride and epoxides catalyzed by a tertiary amine as proposed by Antoon and Koenig. Figure S1: FTIR-ATR spectra of limonene oxide (in red) and Bis-LO (in blue). Figure S2: ^13^C-NMR spectrum of Bis-LO recorded in DMSO *d*6. Figure S3: ^1^H-^13^C HSQC NMR spectrum of Bis-LO recorded in DMSO *d*6. Figure S4: ^1^H-^13^C HMBC NMR spectrum of Bis-LO recorded in DMSO *d*6. Table S1: Properties of the bis-CHO/HMPA/EMI-based and DGEBA/HMPA/EMI-based materials when changing the anhydride/epoxy molar ratio. Table S2: Properties of the bis-CHO/HMPA/EMI-based and DGEBA/HMPA/EMI-based materials when changing the initiator weight percentage. Figure S5: DSC thermograms of: i/ bis-CHO/HMPA/EMI-based thermosets on the left and ii/DGEBA/HMPA/EMI-based thermosets on the right, using varying stoichiometry. Figure S6: DSC thermograms of: i/ bis-CHO/HMPA/EMI-based thermosets on the left and ii/DGEBA/HMPA/EMI-based thermosets on the right, using varying amount of initiator.
