# Supplementary material for: Limonene-Based Epoxy: Anhydride Thermoset Reaction Study
Source: Molecules. 2018 Oct 23;23(11):2739. doi: 10.3390/molecules23112739 (PMC6278336; doi:10.3390/molecules23112739)
Supplement: Supplementary file 1 [file molecules-23-02739-s001.pdf]

Limonene-based epoxy:anhydride thermoset reaction study

Guillaume COUTURE, Lérys GRANADO, Florent FANGET, Bernard BOUTEVIN, Sylvain CAILLOL*

Institut Charles Gerhardt Montpellier - UMR 5253 - CNRS, Université Montpellier, ENSCM – 240 Avenue Emile Jeanbrau, 34296 Montpellier Cedex 5, France.

* Corresponding author: Dr. Sylvain Caillol, Phone: + 33467144327, Email: sylvain.caillol@enscm.fr

Supporting Information

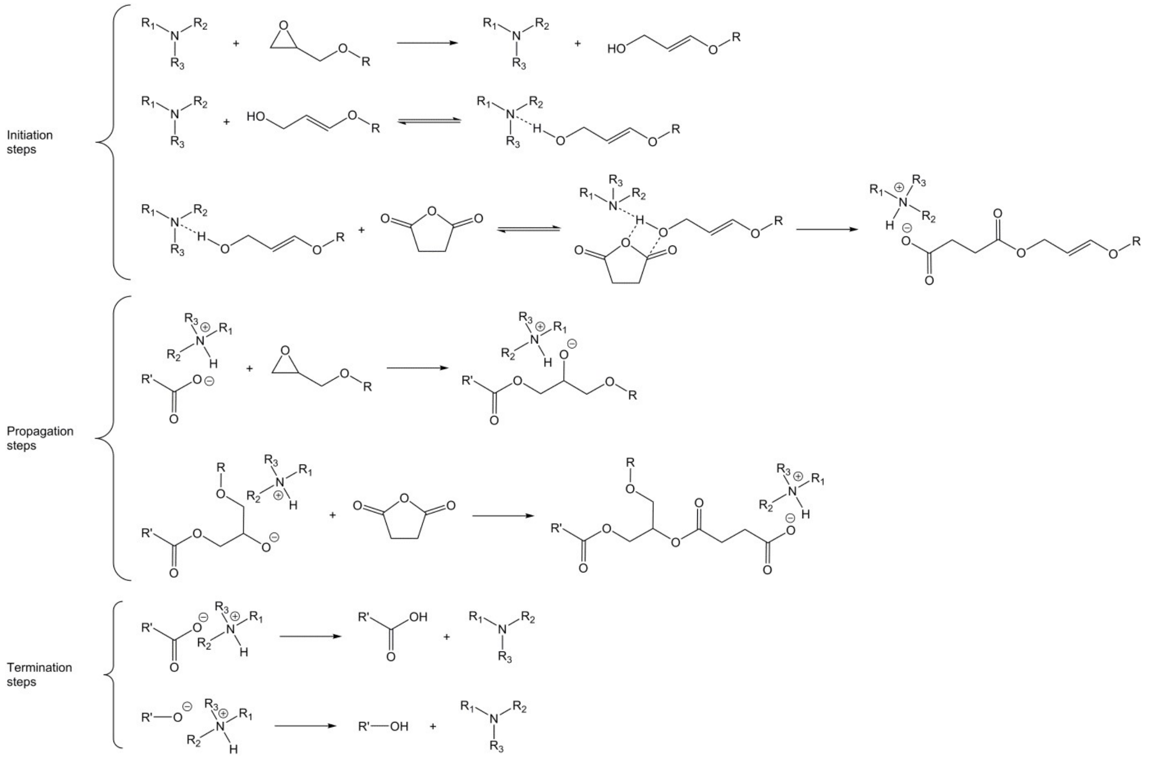

Scheme S 1: Curing mechanism of succinic anhydride and epoxides catalyzed by a tertiary amine as proposed by Antoon and Koenig.

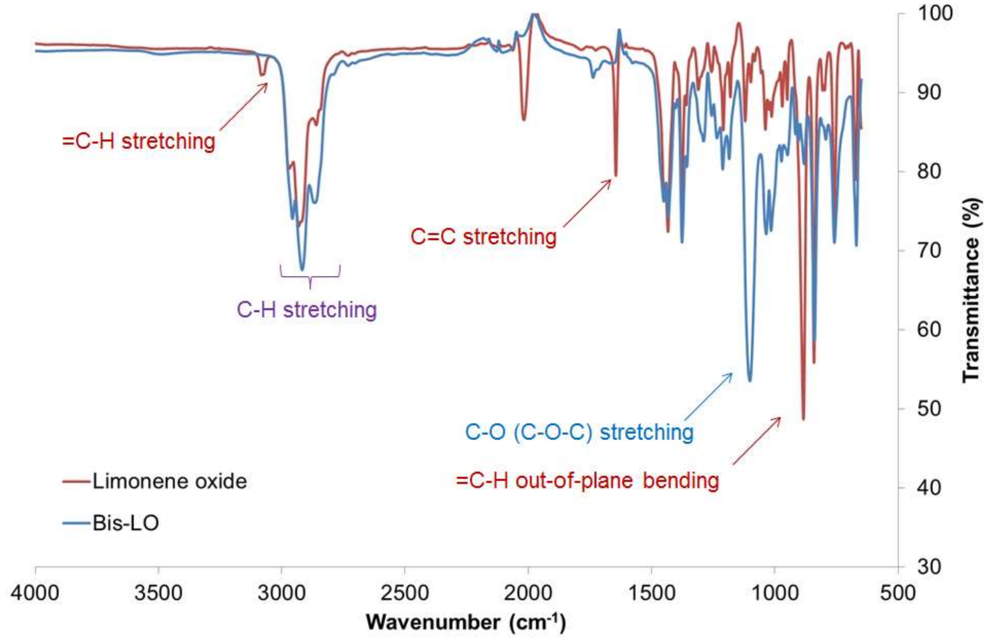

Figure S1: FTIR-ATR spectra of limonene oxide (in red) and Bis-LO (in blue).

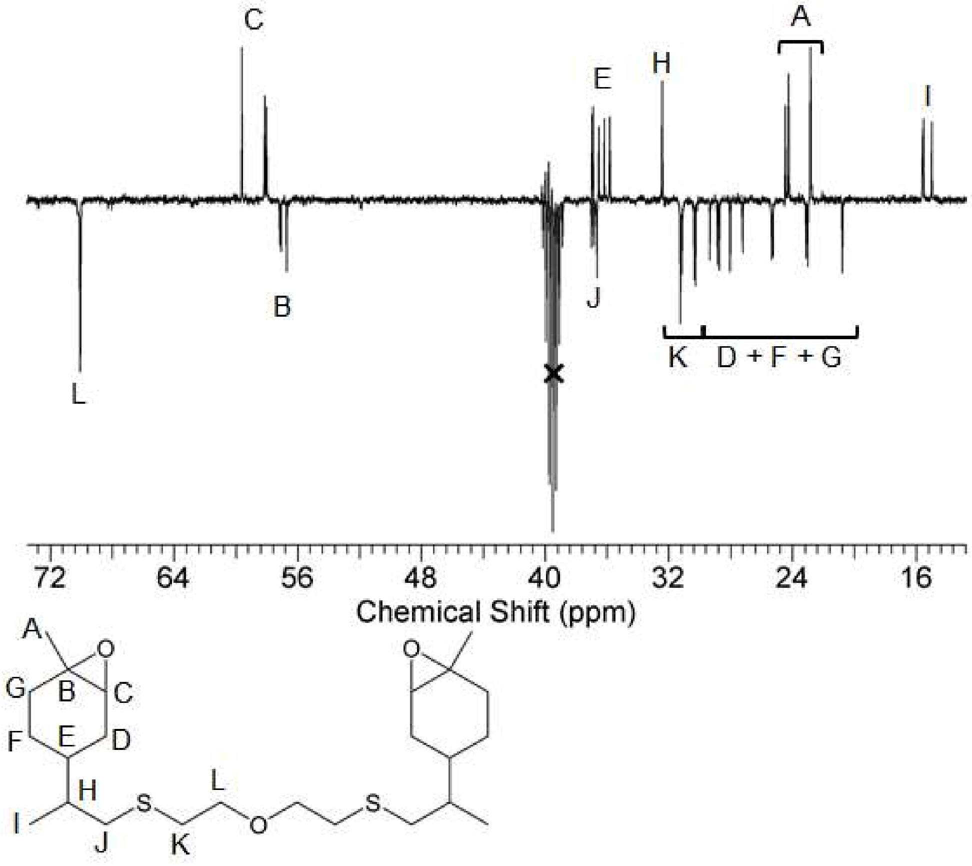

Figure S2 : ^{13}C NMR spectrum of Bis-LO recorded in DMSO d6.

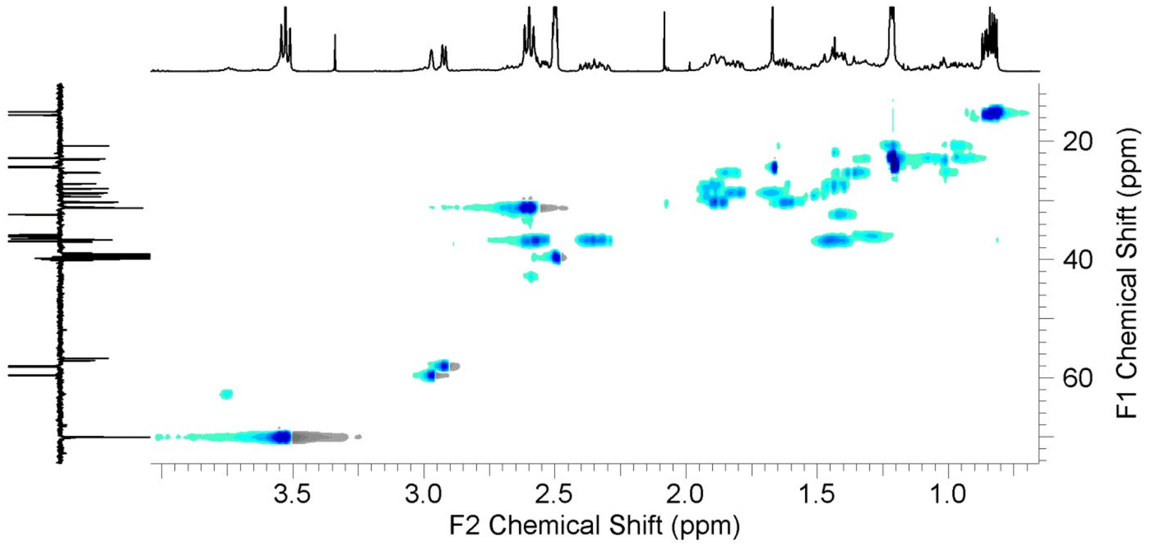

Figure S3: ^{1}H-^{13}C HSQC NMR spectrum of Bis-LO recorded in DMSO d6.

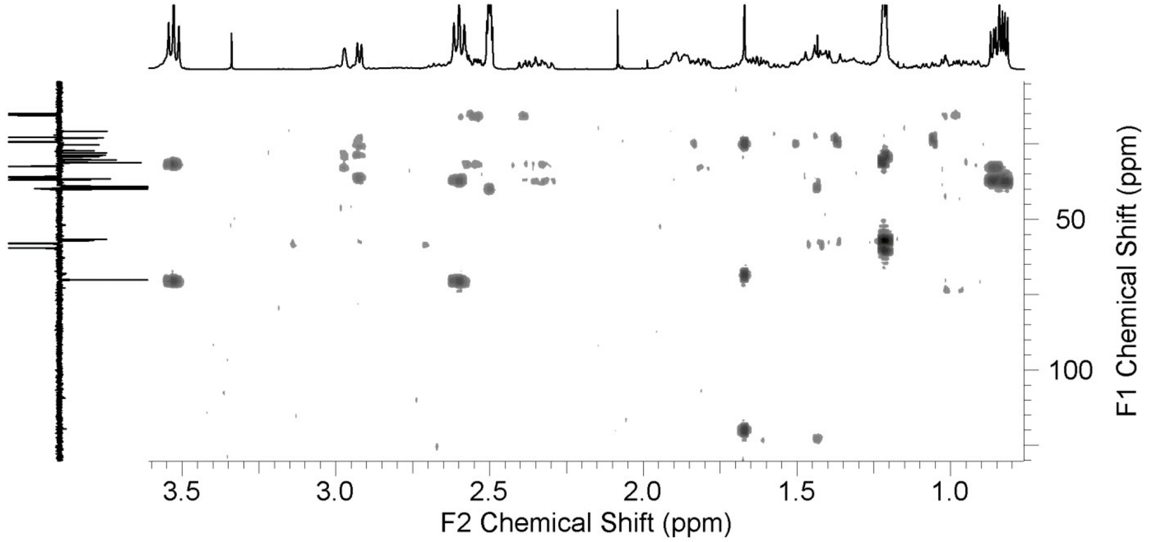

Figure S4: ^{1}H-^{13}C HMBC NMR spectrum of Bis-LO recorded in DMSO d6.

Table S 1: Properties of the bis-CHO/HMPA/EMI-based and DGEBA/HMPA/EMI-based materials when changing the anhydride/epoxy molar ratio.

	bis-CHO/HMPA/EMI					DGEBA/HMPA/EMI						
Sample	bCHO1	bCHO2	bCHO3	bCHO4	bCHO5	D1	D2	D3	D4	D5	D6	D7
Molar ratio Anhydride/ Epoxy	0.76	0.87	0.99	1.18	1.31	0.71	0.80	0.89	0.92	1.11	1.17	1.20
$T_{d,10\%}$ (°C)	316	320	309	328	319	411	406	406	407	409	403	404
T_{max} (°C)	389	388	390	382	382	446	445	446	441	443	438	440
Char (%)	0	0	0	9.6	0	15.0	11.5	6.1	11.2	0	5.6	6.5
T_g (°C)	119	122	117	117	122	155	158	158	156	153	154	152

Table S 2: Properties of the bis-CHO/HMPA/EMI-based and DGEBA/HMPA/EMI-based materials when changing the initiator weight percentage.

	bis-CHO/HMPA							DGEBA/HMPA				
Sample	bCHO6	bCHO7	bCHO8	bCHO9	bCHO10	bCHO11	bCHO12	D8	D9	D10	D11	D12
Initiator wt.%	2.12	3.93	4.05	6.3	7.66	9.29	9.92	1.88	3.91	5.71	8.02	9.85
$T_{d,10\%}$ (°C)	335	306	314	303	301	291	314	409	402	399	382	375
T_{max} (°C)	386	384	382	383	380	384	382	440	441	440	436	430
Char (%)	3.1	0	0	0	5.3	0	0.2	10.2	9.3	12.5	6.6	7.1
T_g (°C)	122	120	116	115	105	107	105	153	146	141	140	133

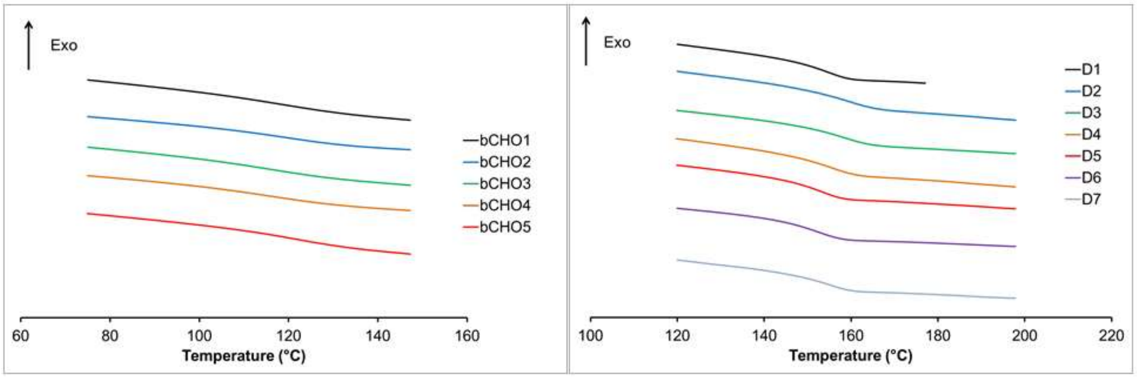

Figure S5: DSC thermograms of : i/ bis-CHO/HMPA/EMI-based thermosets on the left and ii/DGEBA/HMPA/EMI-based thermosets on the right, using varying stoichiometry.

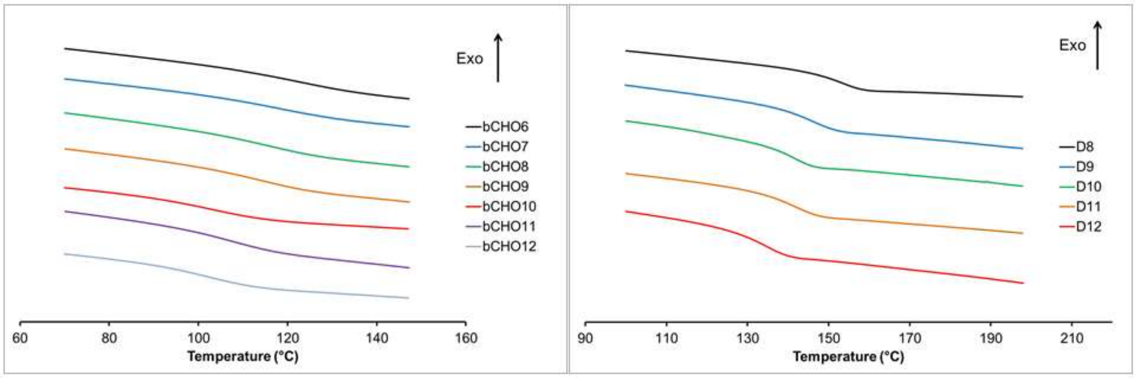

Figure S6: DSC thermograms of : i/ bis-CHO/HMPA/EMI-based thermosets on the left and ii/DGEBA/HMPA/EMI-based thermosets on the right, using varying amount of initiator.
